# African Vegetables (*Clerodendrum volubile* Leaf and *Irvingia gabonensis* Seed Extracts) Effectively Mitigate Trastuzumab-Induced Cardiotoxicity in Wistar Rats

**DOI:** 10.1155/2020/9535426

**Published:** 2020-10-15

**Authors:** Olufunke Olorundare, Adejuwon Adeneye, Akinyele Akinsola, Sunday Soyemi, Alban Mgbehoma, Ikechukwu Okoye, James M. Ntambi, Hasan Mukhtar

**Affiliations:** ^1^Department of Pharmacology and Therapeutics, Faculty of Basic Medical Sciences, College of Health Sciences, University of Ilorin, Ilorin, Kwara State, Nigeria; ^2^Department of Pharmacology, Therapeutics and Toxicology, Faculty of Basic Clinical Sciences, Lagos State University College of Medicine, 1-5 Oba Akinjobi Way, G.R.A., Ikeja, Lagos State, Nigeria; ^3^Department of Pathology and Forensic Medicine, Faculty of Basic Clinical Sciences, Lagos State University College of Medicine, 1-5 Oba Akinjobi Way, G.R.A., Ikeja, Lagos State, Nigeria; ^4^Department of Oral Pathology and Medicine, Faculty of Dentistry, Lagos State University College of Medicine, 1-5 Oba Akinjobi Way, G.R.A., Ikeja, Lagos State, Nigeria; ^5^Department of Nutritional Sciences, College of Agricultural and Life Sciences, University of Wisconsin, Madison, 433 Babcock Drive, Madison, WI 53706-1544, USA; ^6^Department of Dermatology, University of Wisconsin, Madison, Medical Science Center, 1300 University Avenue, Madison, WI 53706, USA

## Abstract

Trastuzumab (*TZM*) is a humanized monoclonal antibody that has been approved for the clinical management of HER2-positive metastatic breast and gastric cancers but its use is limited by its cumulative dose and off-target cardiotoxicity. Unfortunately, till date, there is no approved antidote to this off-target toxicity. Therefore, an acute study was designed at investigating the protective potential and mechanism(s) of *CVE* and *IGE* in *TZM*-induced cardiotoxicity utilizing cardiac enzyme and oxidative stress markers and histopathological endpoints. 400 mg/kg/day *CVE* and *IGE* dissolved in 5% DMSO in sterile water were investigated in Wistar rats injected with 2.25 mg/kg/day/*i.p.* route of *TZM* for 7 days, using serum *cTnI* and LDH, complete lipid profile, cardiac tissue oxidative stress markers assays, and histopathological examination of *TZM*-intoxicated heart tissue. Results showed that 400 mg/kg/day *CVE* and *IGE* profoundly attenuated increases in the serum *cTnI* and LDH levels but caused no significant alterations in the serum lipids and weight gain pattern in the treated rats. *CVE* and *IGE* profoundly attenuated alterations in the cardiac tissue oxidative stress markers' activities while improving *TZM*-associated cardiac histological lesions. These results suggest that *CVE* and *IGE* could be mediating its cardioprotection via antioxidant, free radical scavenging, and antithrombotic mechanisms, thus, highlighting the therapeutic potentials of *CVE* and *IGE* in the management of *TZM*-mediated cardiotoxicity.

## 1. Introduction

Trastuzumab, a humanized monoclonal antibody targeted against epidermal growth factor receptor 2 (HER2), was approved by the United States Food and Drug Administration (FDA) for the clinical management of HER2-positive breast cancers either as an adjuvant or neoadjuvant, and metastatic breast and gastric carcinomas and metastatic gastric cancer [1]. In mediating its cytotoxic action, trastuzumab is known to bind to the domain IV of the extracellular domain of HER2 and triggers cascade tumor-suppressive actions including the activation of antibody-dependent cell-mediated cytotoxicity, inhibition of HER2 extracellular domain cleavage, disruption of HER2 receptor homo- and heterodimerization extracellular segment of HER2 and consequently resulting in the inhibition of HER2-mediated malignant transformation [1, 2]. Trastuzumab use as a key treatment therapy for advanced HER2-positive breast carcinoma has also been reported to have yielded unequivocal improvements in the clinical treatment outcome of this disease [3]. Clinically, trastuzumab is either used alone or in combination with other cytotoxic agents especially with the anthracycline doxorubicin usually in a pegylated form although it is reported to be most effective in its combination form [4] since DOX enters its target cells by simple diffusion, intercalates into DNA, and inhibits topoisomerase II to hinder and completely stall DNA replication [5]. However, wide-scale clinical use of trastuzumab-based therapies has been significantly limited by its adverse cardiac dysfunctions and dilated cardiomyopathy-related congestive heart failures, which have been reported to occur in up to 27% of HER2-positive metastatic breast cancer patients on its combination therapy with doxorubicin [2]. Trastuzumab has been reported to dysregulate HER2 signaling pathways and suppress autophagy by activating autophagy-inhibitory Erk/mTOR/Ulk 1 signaling cascade in cardiomyocytes and overtly resulting in the massive mitochondrial and toxic reactive oxygen species (ROS) accumulation in human cardiomyocytes [6, 7]. As a clinical strategy of preventing the development of trastuzumab-induced cardiotoxicity, Wu et al. [8] recently investigated and reported the clinical efficacy and attenuation of trastuzumab-induced cardiac dysfunction in HER2-positive breast cancer patients using fixed 440 mg dose monthly administration of trastuzumab. Unfortunately, till date, there are no approved effective therapeutic agent(s) available that could prevent the development of this unwanted/adverse effect of trastuzumab without comprising its efficacy.

*Clerodendrum volubile* P. Beauv (known as White butterfly in English language) is a climbing and edible West African vegetable, belonging to the *Verbenaceae* family [9] but was recently reclassified to as belonging to the *Labiatae* family [10]. In the Niger-Delta region of Nigeria where the plant is predominantly cultivated for consumption wholly as green leafy vegetable or as food condiment to improve soup taste, it is used for the local management of gouty arthritis, rheumatism, dropsy, swellings/edema, and ulcers [9, 11]. Phytochemically, *Clerodendrum volubile* leaf extracts have been reported to contain secondary metabolites such as alkaloids, flavonoids, saponins, anthraquinone, and cardiac glycoside [12]. The phenolic-rich solvent fractions of the plant extract have been reported to elicit antihyperglycemic activity through *α*-amylase, *α*-glucosidase, and improvement in the glucose tolerance while its antihypertensive activity was mediated via angiotensin I converting enzyme inhibition [9, 10]. Similarly, the antioxidative, immunomodulatory, anti-inflammatory, and cytotoxic activities of the plant have also been reported [12–15]. *Clerodendrum volubile* is reported to be very rich in polyphenols (especially flavonoids) content which is conferred on its potent antioxidant potential [9, 16, 17].

*Irvingia gabonensis* (Aubry-Lecomte ex O'Rorke) Bail belonging to the family, Irvingiaceae, is known as African Mango (in English). Its common English names include bread tree, African wild mango, wild mango, and bush mango [18, 19], while its local names in Nigeria include “Apon” and “Ogbono” (amongst the Yoruba, Southwest Nigeria and Igbo, Southeast Nigeria, respectively). *Irvingia gabonensis* is widely cultivated in West African countries including southwest and southeast Nigeria, southern Cameroon, Côte d'Ivoire, Ghana, Togo, and Benin, to produce its edible fruit whose seed is used in the preparation of local delicious viscous soup for swallowing yam and cassava puddings [20]. Fat extracted from its seeds is commonly known as dika fat and majorly consists of C12 and C14 fatty acids, alongside with smaller quantities of C10, C16 and C18, glycerides, and proteins [20]. *Irvingia gabonensis* seeds are also a good source of nutrients including a variety of vitamins and minerals such as sodium, calcium, magnesium, phosphorus, and iron. It is also a rich source of flavonoids (quercetin and kaempferol), ellagic acid, mono-, di-, and tri-O-methyl-ellagic acids, and their glycosides which are potent antioxidants [21, 22]. Phytochemical analysis of its seeds showed that it contains tannins, alkaloids, flavonoids, cardiac glycosides, steroids, carbohydrate, volatile oils, and terpenoids [23–25] and its proximate composition of moisture 1.4 ± 0.11%, ash 6.8 ± 0.12%, crude lipid 7.9 ± 0.01%, crude fibre 21.6 ± 0.45%, and crude protein 5.6 ± 0.20% [25]. Similarly, proximate analysis of its soup shows that it contains 9% protein, 70.42% fat, 4.61% fibre, 1.92% ash, and 11.91% carbohydrate [26]. Specific compounds already isolated from the seed extract of include: methyl 2- [2-formyl-5-(hydroxymethyl)-1 H-pyrrol1yl]-propanoate, kaempferol-3-0-*β*-D-6^″^ (p-coumaroyl) glucopyranoside and lupeol (3*β*-lup-20(29)-en-3-ol with lupeol exhibiting the most abundant with the most significant antioxidant activities [27].

In the absence of any clinically approved chemotherapeutic or chemoprophylactic agents for the clinical management of trastuzumab-induced cardiovascular events, the current study was designed at investigating possible ameliorative potential of the ethanol extracts of *Clerodendrum volubile* leaves and *Irvingia gabonensis* seeds in trastuzumab-induced cardiotoxicity in Wistar rats intraperitoneally injected with 2.25 mg/kg/day of trastuzumab for 7 days. The effects of oral pretreatments with 400 mg/kg/day of *Clerodendrum volubile* ethanol leaf extract as well as 400 mg/kg/day of *Irvingia gabonensis* ethanol seed extract were investigated in trastuzumab intoxicated rat hearts using cardiac enzyme biomarkers such as cardiac troponin I (*cTnI*) and cardiac lactate dehydrogenase (LDH), complete lipid profile, cardiovascular disease risk indices (atherogenic index (AI) and coronary artery disease risk index (CRI)), oxidative stress markers, as well as the histopathological studies of the trastuzumab-treated cardiac tissues as measuring endpoints for the study.

## 2. Materials and Methods

### 2.1. Plant Materials

Stock of fresh mature whole plants of *Clerodendrum volubile* and fresh seeds of *Irvingia gabonensis* were purchased from Herbal Vendors in Isikan Market in Akure, Ondo State, Nigeria, in the month of February 2020. Samples of the *Clerodendrum volubile* plant obtained were subjected to botanical identification and referencing at the University of Ilorin (UNILORIN) Herbarium with a voucher specimen number: UIL/001/2019/1254 as previously reported by Akinsola (2019) [28]. Fresh leaves, inflorescence, and fruits of *Irvingia gabonensis* were equally processed for botanical identification and authentication and voucher specimen with reference number (UIL/001/2019/1364) was also deposited in UNILORIN Herbarium.

### 2.2. Extraction Process

Fresh leaves of *Clerodendrum volubile* were destalked from the whole plant, then gently but thoroughly rinsed under running tap water and completely air-dried at the room temperature (28-33°C) until the weight of the dried leaves was constant. The dried leaves were then pulverized using Milling Machine and kept in water- and air-tight containers.

1.50 kg of the pulverized leaves was completely macerated in 8 liters of absolute ethanol at room temperature for 5 days but intermittently shaken to ensure complete dissolution. Thereafter, the solution was first filtered with cotton wool and then 110 mm Whatman filter paper. The resultant filtrate was then concentrated *in vacuo* using a rotary evaporator (B˙U˙CHI Rotavapor® Model R-215, Switzerland) with Vacuum Module V-801 EasyVac®, Switzerland) set at a revolution of 70 rpm and a temperature at 36°C before it was completely dried over a water bath preset at 40°C. The jelly-like, dark-colored residue left behind was weighed, stored in air- and water-proof container which was kept in a refrigerator at 4°C. From this stock, fresh solutions were made whenever required.

%Yield was calculated as = (weight of crude extract obtained (*g*)/weight of starting pulverized dry leaf extracted (*g*)) × 100.

The same procedure was performed with 1.5 kg of the pulverized, dried seeds of *Irvingia gabonensis*.

### 2.3. Experimental Animals

Young adult male Wistar Albino rats (aged 8-12 weeks old and body weight: 150-190 g) used in this study were obtained from the Animal House of the Lagos State University College of Medicine, Ikeja, Lagos State, Nigeria, after an ethical approval (UERC Approval number: UERC/ASN/2020/2072) was obtained from the University of Ilorin Ethical Review Committee for Postgraduate Research. The rats were handled in accordance with international principles guiding the Use and Handling of Experimental Animals [29]. The rats were maintained on standard rat feed (Ladokun Feeds, Ibadan, Oyo State, Nigeria) and potable water which were made available *ad libitum*. The rats were maintained at an ambient temperature between 28-30°C, humidity of 55 ± 5%, and standard (natural) photoperiod of approximately 12/12 hours of alternating light and dark periodicity.

### 2.4. Measurement of Body Weight

The body weights of rats were taken on days 1 and 7 of the experiment and determined on a digital rodent weighing scale (®Virgo Electronic Compact Scale, New Delhi, India). The obtained values were expressed in grams (g).

### 2.5. Induction of Trastuzumab- (TZM-) Induced Cardiotoxicity and Other Drug Treatment of Rats

Prior to commencement of the experiment, rats were randomly allotted into 7 groups of 7 rats per group such that the weight difference between and within groups was not more than ±20% of the average weight of the sample population of rats used for the study. However, the choice of the therapeutic dose range of 400 mg/kg/day of *CVE* and *IGE* was made based on the results of the preliminary studies conducted.

In this experimental repeated-dose model, Group I rats which served as untreated control were orally pretreated with 10 ml/kg/day of sterile water but equally treated with 1 ml/kg/day of sterile water and administered via intraperitoneally for 7 days. Group II and III rats were orally treated with 400 mg/kg/day of *CVE* and *IGE* dissolved in 5% DMSO sterile water (*CVE* and *IGE* being only partly soluble in water and DMSO an organosulfur polar aprotic and inert solvent that readily dissolves both polar and nonpolar compounds) but treated with 1 ml/kg/day of sterile water and administered intraperitoneally for 7 days, respectively. Group IV rats were orally pretreated with 10 ml/kg/day of sterile water 3 hours before intraperitoneal injection of 2.25 mg/kg/day of *TZM* (®CAMMab, Biocon Limited, Km 34 Tumkur Road, T-Bengur, Nelamangala Taluk, Bangalore-56 123, India) dissolved in accompanying sterile water for 7 days. Group V rats which served as the positive control group were equally pretreated with 20 mg/kg/day of Vitamin C 3 hours before treatment with 2.25 mg/kg/day of *TZM* dissolved in sterile water administered intraperitoneally for 7 days. Group VI and VII rats were orally pretreated with 400 mg/kg/day of *CVE* and *IGE* 3 hours before treatment with 2.25 mg/kg of *TZM* dissolved in sterile water and administered intraperitoneally daily for 7 days (Table 1). The choice of vitamin C was made being a standard antioxidant agent, and its effect as positive control was compared with other treatment groups. The dose of *TZM* adopted was as described by Poon et al. [30] and Riccio et al. [31].

### 2.6. Blood Sample Collection

On the 7^th^ day which was the last day of the experiment, the rats were weighed and later fasted overnight but drinking water was made available *ad libitum*. On the 8^th^ day, fasted rats were sacrificed and whole blood samples were collected directly from the heart under inhaled diethyl ether anesthesia. Blood samples were carefully collected with a fine 21G Needle and 5 ml Syringe (Hangzhou Longde Medical Products Co. Ltd., Hangzhou, China) without causing damage to the heart tissues. The rat heart, liver, and kidneys were identified, harvested *en bloc*, and weighed on a digital weighing scale.

### 2.7. Biochemical Assays

Blood samples obtained directly from the heart chamber were allowed to clot and then centrifuged at 5000 rpm to separate clear sera from the clotted blood samples. The clear samples were obtained for assays of the following biochemical parameters: serum cardiac troponin I, LDH, TG, TC, and cholesterol fractions (HDL-c, LDL-c, and VLDL-c). Serum lipids were assayed using methods of Tietz [32] while serum *cTnI* and LDH were estimated standard bioassay procedures.

### 2.8. Calculation of AI and CRI

AI was calculated as LDL − c (mg/dl) ÷ HDL − c (mg/dl) [33] while CRI was calculated as TC (mg/dl) ÷ HDL − c (mg/dl) [34].

### 2.9. Determination of Antioxidant Activities in the Rat Cardiac Tissues

After the rats were sacrificed humanely under inhaled diethyl ether, the heart was harvested *en bloc*. The heart was gently and carefully divided into two halves (each consisting of the atrium and ventricle) using a new surgical blade. The left half of the heart was briskly rinsed in ice-cold 1.15% KCl solution in order to preserve the oxidative enzyme activities of the heart before being placed in a clean sample bottle which itself was in an ice-pack filled cooler. This is to prevent the breakdown of the oxidative stress enzymes in these organs.

#### 2.9.1. Determination of SOD Activities in the Heart Tissues

Superoxide dismutase activity was determined by its ability to inhibit the autooxidation of epinephrine by the increase in absorbance at 480 nm as described by Paoletti et al. [35]. Enzyme activity was calculated by measuring the change in absorbance at 480 nm for 5 minutes.

#### 2.9.2. Determination of CAT Activities in the Heart Tissues

Tissue CAT activities were determined by the method described by Hadwan [36]. The specific activity of CAT was expressed as U/ml.

#### 2.9.3. Determination of GSH, GPx, and GST Activities in the Heart Tissue

The reduced glutathione (GSH) content in the heart tissue was estimated according to the method described by Rahman et al. [37]. To the homogenate, 10% TCA was added and centrifuged. One millilitre of the supernatant was treated with 0.5 ml of Elman's reagent (19.8 mg of 5,5-dithiobisnitro benzoic acid (DTNB) in 100 ml of 0.1% sodium nitrate) and -3.0 ml of phosphate buffer (0.2 M, pH 8.0). The absorbance was read at 412 nm. Similarly, GPx and GST activities were determined using the method of Faraji et al. [38] and Vontas et al. [39].

#### 2.9.4. Determination of MDA Activities in the Heart Tissues

The method of Buege and Aust [40] was adopted in determining MDA activities in the cardiac tissue. One millilitre of supernatant was added to 2 ml of (1 : 1 : 1 ratio) TCA-TBA-HCl reagent (thiobarbituric acid 0.37%, 0.24 N HCl, and 15% TCA) tricarboxylic acid, thiobarbituric acid, reagent boiled at 100°C for 15 minutes, and allowed to cool. Flocculent material was removed by centrifuging at 3000 rpm for ten minutes. The supernatant was removed, and the absorbance was read at 532 nm against a blank. MDA was calculated using the molar extinction for MDA-TBA-complex of 1.56 × 10^5^ m^−1^ cm^−1^.

#### 2.9.5. Histopathological Studies of the Heart

Using the remaining equally divided harvested heart, the right halves of the seven randomly selected rats from each treatment and control groups were subjected to histopathological examinations, the right ventricle being the most susceptible to doxorubicin toxicity of the heart chambers. After rinsing in normal saline, the dissected right half of was preserved in 10% formo-saline before it was completely dehydrated in absolute (100%) ethanol. It was then embedded in routine paraffin blocks. From the embedded paraffin blocks, 4-5 *μ*m thick sections of the tissue was prepared and stained with hematoxylin-eosin stain. These were examined under a photomicroscope (Model N-400ME, CEL-TECH Diagnostics, Hamburg, Germany) connected with a host computer. Sections were illuminated with white light from a 12 V halogen lamp (100 W) after filtering with a 520 nm monochromatic filter. The slides were examined for associated histopathological lesions [41].

### 2.10. Statistical Analysis

Data were presented as mean ± S.D. and mean ± S.E.M. of seven observations for the body weight and biochemical parameters, respectively. Statistical analysis was done using a two-way analysis of variance followed by post hoc test, Student-Newman-Keuls test on GraphPad Prism Version 5. Statistical significance was considered at *p* < 0.05, *p* < 0.01, and *p* < 0.001.

## 3. Results

### 3.1. %Yield

Complete extraction of the pulverized dry leaves *Clerodendrum volubile* in absolute ethanol was calculated to be 8.39%. The resultant residue was a dark color, sticky and jelly-like, sweet-smelling (bland) residue which was not completely soluble in water but completely soluble in methanol and ethanol. Similarly, complete extraction of *Irvingia gabonensis* ethanol seed extract in absolute ethanol resulted in a yield of 58%, which was a dark brown oily and aromatic residue that was only soluble in methanol and ethanol.

### 3.2. Effect of CVE and IGE on the Average Body Weight of TZM-Treated Rats

Table 2 shows the effect of repeated daily intraperitoneal injection with 2.25 mg/kg of *TZM* and oral pretreatments with 20 mg/kg/day of vit. C and 400 mg/kg/day of *CVE* and *IGE*, respectively, on the average body weight on days 1 and 7, percentage weight change (%∆wt.), and relative heart weight of treated rats. Repeated intraperitoneal *TZM* injection did not significantly alter (*p* > 0.05) the weight gain pattern and relative heart weight in the *TZM* only treated (Group IV) rats when compared to untreated control (normal) rats (Group II) as well as *CVE*- (Group VI) and *IGE*- (Group VII) pretreated rats (Table 2). Similarly, vit. C pretreatment did not significantly alter the weight gain pattern and relative heart weight in the *TZM*-treated rats (Table 2).

### 3.3. Effect of CVE and IGE on Cardiac Marker Enzymes (LDH and cTnI) of TZM-Treated Rats

Repeated daily intraperitoneal *TZM* injection for 7 days resulted in significant increases (*p* < 0.0001) in the serum LDH and *cTnI* levels when compared to that of untreated negative (control) (Group I) values (Table 3). However, 400 mg/kg/day of *CVE* and *IGE* oral pretreatments significantly attenuated (*p* < 0.0001) increases in the serum LDH and *cTnI* levels (Table 3). Similarly, 20 mg/kg/day of vit. C pretreatment also significantly (*p* < 0.001 and *p* < 0.0001) attenuated increases in the serum LDH and *cTnI* though at a lower level of statistical significance when compared to either *CVE* or *IGE* (Table 3).

### 3.4. Effect of CVE and IGE on the Serum Lipids (TG, TC, HDL-c, LDL-c, and VLDL-c) Level of TZM-Treated Rats

Repeated *TZM* intraperitoneal injections did not cause significant (*p* > 0.05) alterations in the serum lipids measured when compared to the untreated control (Group I) values (Table 4). However, repeated daily oral pretreatments with 400 mg/kg/day of *CVE* and *IGE* resulted in insignificant reductions in the serum levels of TG, TC, HDL-c, LDL-c, and VLDL-c when compared to *TZM* only-treated rats (Table 4). Similarly, vit. C did not cause significant (*p* > 0.05) alterations in the serum TG, TC, LDL-c, and VLDL-c levels when compared to *TZM* only-treated rats (Table 4).

### 3.5. Effect of CVE and IGE on the Atherogenic Index (AI) and Coronary Artery Disease Index (CRI) of TZM-Treated Rats

Repeated intraperitoneal injections with 2.25 mg/kg/day of *TZM* to treated rats resulted in an insignificant (*p* > 0.05) increase in the AI and CRI values when compared to the untreated control (Group I), *CVE* only treated (Group II), and *IGE* only treated (Group III) values (Table 5). Oral pretreatments with 400 mg/kg/day of *CVE* and *IGE*, however, resulted in insignificant (*p* > 0.05) reductions in the AI and CRI values when compared to *TZM* only-treated rats (Table 5). Similar insignificant reductions (*p* > 0.05) in the AI and CRI values were caused by 20 mg/kg/day of vit. C oral pretreatment (Table 5).

### 3.6. Effect of CVE and IGE on the Cardiac Tissue Oxidative Stress Markers (GSH, GST, GPx, SOD, CAT, and MDA) of TZM-Treated Rats

Repeated *TZM* intraperitoneal injection to treated rats resulted in significant attenuation (*p* < 0.05 and *p* < 0.0001) in SOD, CAT, GST activities, and GSH levels while there were significant increases (*p* < 0.0001) in the GPx and MDA activities (Table 6). However, repeated oral treatments with 400 mg/kg/day of *CVE* and *IGE* significantly (*p* < 0.001 and *p* < 0.0001) attenuated the alterations in the activities of these oxidative stress markers in the cardiac tissue restoring their activities to normal as recorded for Groups I-III values. These values were also comparable to those of vit. C-treated group (Table 6).

### 3.7. Histological Effect of CVE and IGE on TZM-Treated Heart

**Figure 1:**
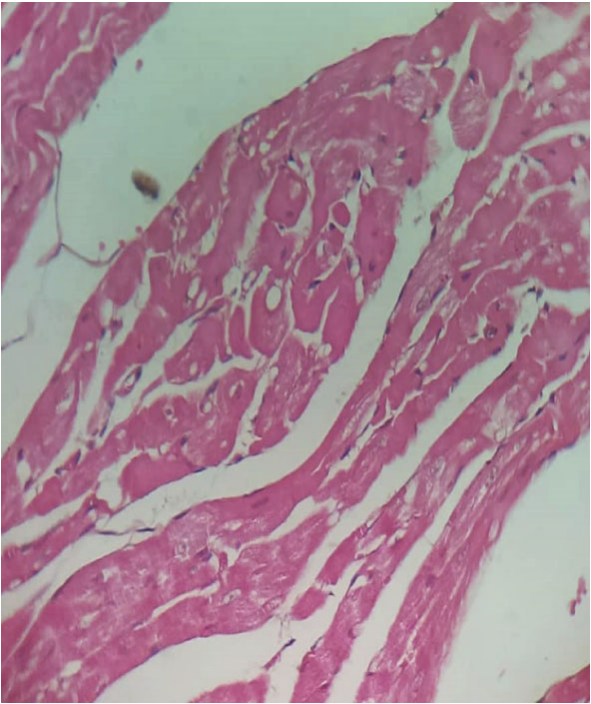
A cross-sectional representative of *TZM* intoxicated rat heart pretreated with 10 ml/kg/day of sterile water showing severe vascular congestion and intraparenchymal hemorrhage as well as coronary arterial wall thickening with endothelial microthrombi formation indicative of coronary arteriosclerosis (x400 magnification, hematoxylin-eosin stain).

**Figure 2:**
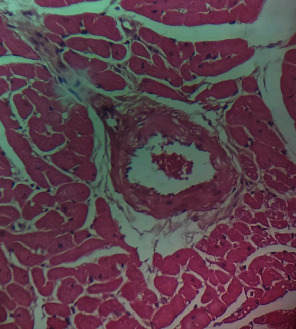
A cross-sectional representative of the normal rat heart showing normal cardiac histoarchitecture (x400 magnification, hematoxylin-eosin stain).

**Figure 3:**
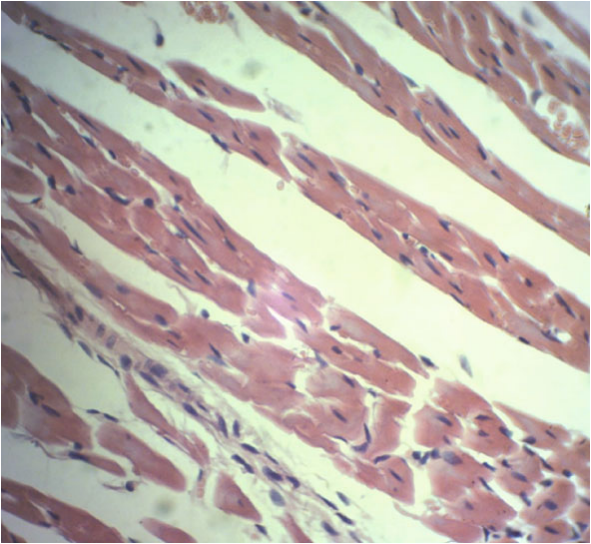
A cross-sectional representative of the 400 mg/kg/day of *CVE* treated-rat heart showing normal cardiac histoarchitecture with mild pericardiac fat deposit (x400 magnification, hematoxylin-eosin stain).

**Figure 4:**
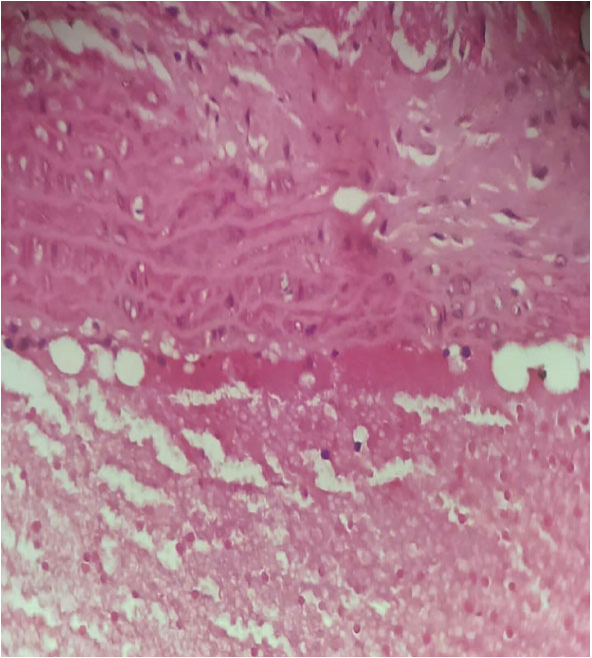
A cross-sectional representative of the 400 mg/kg/day of *IGE* treated-rat heart showing normal cardiac histoarchitecture (x400 magnification, hematoxylin-eosin stain).

**Figure 5:**
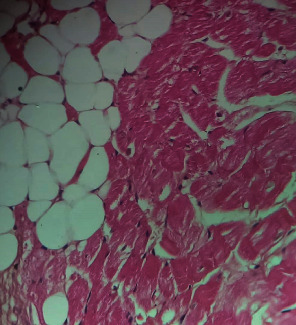
A photomicrograph of cross-sectional representative of *TZM* intoxicated rat heart orally pretreated with 20 mg/kg/day of vit. C showing mild vascular congestion, mild intraparenchymal hemorrhage, and increased pericardial fat thickness (x400 magnification, hematoxylin-eosin stain).

**Figure 6:**
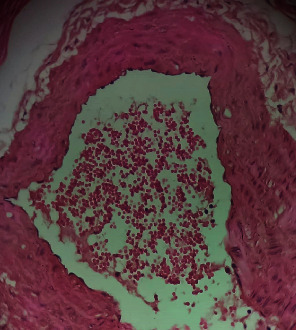
A photomicrograph of cross-sectional representative of *TZM* intoxicated rat heart treated with 400 mg/kg/day of *CVE* showing mild intraparenchymal hemorrhage with thickened coronary arterial wall suggestive of coronary arteriosclerosis (x400 magnification, hematoxylin-eosin stain).

**Figure 7:**
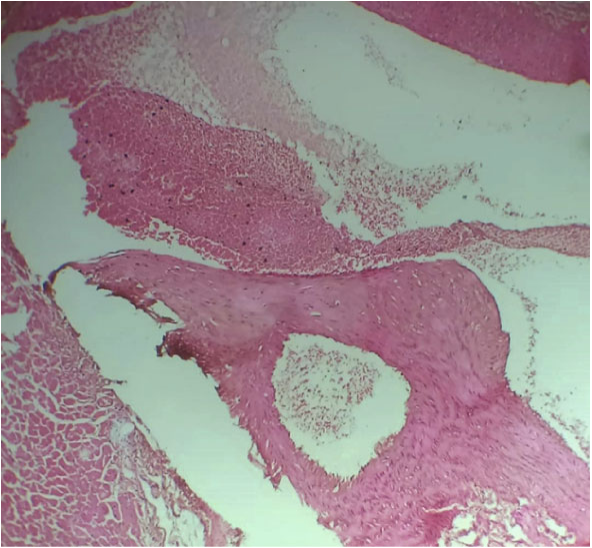
A photomicrograph of cross-sectional representative of *TZM* intoxicated rat heart treated with 400 mg/kg/day *IGE* showing mild vascular congestion and coronary artery recanalization (x100 magnification, hematoxylin-eosin stain).

Repeated intraperitoneal injections of rats with 2.25 mg/kg/day of *TZM* for 7 days resulted in marked vascular congestion, intraparenchymal hemorrhage, and coronary artery microthrombi formation with the preservation of the cardiac myocyte cytoarchitecture (Figure 1). This is in sharp contrast with normal coronary artery and cardiomyocyte architecture recorded for Groups I-III cardiac muscle that were orally treated with 10 ml/kg/day of sterile water, 400 mg/kg/day of *CVE*, and 400 mg/kg/day of *IGE* only, respectively, with no remarkable histological changes in the treated heart muscles (Figures 2–4). However, repeated oral pretreatments with 20 mg/kg/day of vit. C (standard antioxidant drug), 400 mg/kg/day of *CVE*, and 400 mg/kg/day of *IGE* markedly improved *TZM*-induced coronary artery histopathological alterations (Figures 5–7) with coronary artery recanalization recorded in *IGE* pretreated, *TZM*-treated (Group VII) rats (Figure 7).

## 4. Discussion

Trastuzumab either used alone or in combination with other agents from other classes of cytotoxic agents has remained a cornerstone and key strategy in the clinical management of patients with metastatic breast carcinoma overexpressing the HER2 protein [42, 43]. Despite its wide application in this regard, its clinical use has been limited by its cumulative dose-limiting but reversible cardiotoxicity which manifests as a life-threatening dilated cardiomyopathy and congestive cardiac failure [43, 44]. Unfortunately, till date, there are no approved effective chemotherapeutic/chemoprophylactic options available in its amelioration despite efforts being directed towards developing an effective therapeutic alternative, one of which is the antianginal agent, ranolazine, which has been reported to blunt trastuzumab cardiotoxicity mediated via redox-mediated mechanisms [31]. However, ranolazine's clinical use is known to be limited by its serious side effects such as bradycardia, syncope attacks, hematuria, acute renal failure, and its predilection to liver cirrhosis [45, 46]. Therefore, this study investigated the ameliorative potential of *CVE* and *IGE* in *TZM*-related cardiotoxicity in experimental rats. In doing this, experimental *TZM* cardiotoxicity was reliably induced in the treated rats following repeated daily intraperitoneal injection of 2.25 mg/kg of *TZM* for 7 days as evidenced by profound elevations in the serum cardiac markers (*cTnI* and LDH), alterations in the serum lipids profile and cardiovascular disease risk indices, and marked alteration in the oxidative stress markers. All of these biochemical changes were corroborated by remarkable histological lesions such as vascular congestion, intraparenchymal hemorrhage, coronary artery endothelial thickening, and thrombi formation. *cTnI* and LDH are considered reliable markers of cardiotoxicity and are as such used in monitoring drug-induced-cardiotoxicities including *TZM* [47–52]. The fact that the serum levels of *cTn* I and LDH were significantly elevated following repeated administration for 7 days is a strong indication that *TZM*-induced cardiac damage was reliably established and in consonance with reports of other studies [49, 51, 53]. However, repeated oral pretreatments with vitamin C, *CVE*, and *IGE* profoundly attenuated elevations in serum levels of these cardiac markers, thus, indicating the potential therapeutic role of these agents in mitigating the deleterious effects of *TZM* on the integrity cardiac myocytes.

Another significant finding of this study is the effect of *TZM* treatment on the circulating lipids levels. Prolonged *TZM* treatment was also being documented to be associated with dyslipidemia which is characterized by significant increases in the serum triglycerides, very low-density lipoprotein cholesterol (VLDL-c), and low-density lipoprotein cholesterol (LDL-c) [54, 55]. The findings of our study are in agreement with this assertion although *TZM* treatment for 7 days in our study was associated with slight improvements in the circulating lipids levels as well as the cardiovascular disease risk indices. The variance between our result of study and other studies could have resulted from the short duration of *TZM* treatment. This remains a hypothesis until validated by similar studies of longer duration. In the same vein, neither *TZM* treatment nor extracts pretreatment treatment causes any significant changes in the weight gain pattern of the treated rats. Again, it is possible that the short duration of the studies could be responsible for this.

*TZM* like other anticancer agents such as cisplatin has been reported to cause “acute coronary syndrome” which may manifest as coronary ischemia from coronary artery endothelial thrombi and profound elevation in cardiac enzymes which are often prevented with aspirin and intensive anti-ischemic medication with nitrates and *β*-blockers [56]. Acute coronary syndrome is believed to equally result from attendant vascular endothelial dysfunction of the coronary artery and peripheral vasculature, and this endothelial dysfunction is considered an early indicator of atherosclerosis [57, 58]. The histological findings of increased coronary artery endothelial thickening and microthrombi in the *TZM*-only treated rat hearts are indicative of the full experimental induction of *TZM*-related arteriosclerosis and *TZM*-induced cardiotoxicity. Vitamin C has previously been reported to improve endothelial function of conduct arteries in patients with chronic cardiac failure [59]. However, the fact that oral pretreatments with *CVE* and *IGE* effectively improved these histological lesions is strongly reflective of the therapeutic potential effects of these extracts against *TZM*-associated endothelial dysfunction.

Oxidative stress (the shift in the balance between oxidants and antioxidants in favor of oxidants) is the net result of an imbalance between ROS production and destruction (the latter being regulated by antioxidant defense system) [60, 61]. ROS (free radicals and non-radicals) are produced from molecular oxygen as a result of normal cellular metabolism and the 3 major ROS that are of physiological significance are superoxide anion (O_2_^−.^), hydroxyl radical (•OH), and hydrogen peroxide (H_2_O_2_) [60]. Oxidative stress is a consequence of an increased generation of these free radicals and/or reduced physiological activity of antioxidant defenses against free radicals. In containing the activities of the ROS, the body system has evolved an innate antioxidant system to mitigate the possible deleterious effects of oxidative stress on the body organs/systems [60, 62, 63]. The antioxidant systems are basically of two types, namely, enzymatic antioxidants which include SOD, CAT, GSH Px, GSTs, and heme oxygenase-1 and nonenzymatic antioxidants which include vitamins (vitamins C and E), *β*-carotene, uric acid, and GSH, a tripeptide (l-*γ*-glutamyl-l-cysteinyl-l-glycine) that comprise a thiol (sulfhydryl) group (e.g., thioredoxin-1 (Trx-1)) [60, 64]. These antioxidant systems are known to mediate their antioxidant activities via several mechanisms which include the inhibition of free radical formations; protection of cells against apoptosis by interacting with proapoptotic and antiapoptotic signaling pathways; regulation and activation of several transcription factors, such as AP-1, NF-*κ*B, and Sp-1; superoxide and oxygen-free radical scavenging activities [65–70]. Pleiotropic deleterious effects of oxidative stress are observed in numerous disease states and are also implicated in a variety of drug-induced toxicities. Identifiable drugs are alkylating anthracycline antineoplastic agents (doxorubicin), antiretroviral (azidovudine), anti-inflammatory (diclofenac), platinum-based antineoplastic agent (cisplatin), antipsychotic (chlorpromazine) [71], and most recently, a HER2 directed monoclonal antibody (trastuzumab) [7, 72]. However, the effectiveness of conventional cytotoxic drugs is largely based on the generation of ROS and consequently on the increase of oxidative stress that exceeds the reduction capacity of cancerous tissue, resulting in apoptotic cell death [73], and most of the adverse effects emanating from chemotherapy result from excess ROS production in healthy tissues, such as anthracycline-mediated cardiotoxicity, and nephrotoxicity triggered by platinum compounds [74, 75] which are mainly based on the interaction of OH^•^ with target tissue DNA [76, 77]. *TZM* has been reported to potentiate cardiomyocyte toxicity through a “dual-hit” mechanism, which includes alterations in antiapoptotic signalling pathways in cardiomyocytes, inhibition of the neuregulin-1 survival signaling pathway, and angiotensin II-induced activation of NADPH oxidase, with the ability to further increase reactive oxygen species production, ultimately resulting in dilated cardiomyopathy [78, 79].

The present study showed that *TZM* had significant effects on the oxidative stress markers such as SOD, CAT, GST, and GSH whose activities and levels in the treated cardiac tissues were suppressed while the cardiac tissue activities and levels of GPx and MDA were profoundly elevated. These results are similar to others previously reported [31, 80, 81]. *TZM* induces cardiomyocyte toxicity through a mitochondrial pathway depending on ROS production and oxidative stress. *TZM* activates proapoptotic proteins such as *Bax* and induces *mPTP* opening, and these eventually result in mitochondrial defects and dysfunctions [82]. Classes of conventional drugs such as angiotensin-converting enzyme inhibitor (ACEI), angiotensin receptor blocker (ARB), mineralocorticoid receptor antagonist (MRA), nonsteroidal anti-inflammatory drug (NSAID), and lecithinized human recombinant superoxide dismutase (PC-SOD) have been reported to offer cardioprotection against DOX-mediated cardiotoxicities [83]. Natural antioxidant supplements such as coenzyme Q10 [84] and N-acetylcysteine (administered either alone or with vitamins E and C) [85] have been reported to mitigate anthracycline- (doxorubicin-) mediated left ventricular dysfunction and remodeling while melatonin [86] and levocarnitine [87] have also been tested in the clinical setting with positive results. Similarly, plant-derived small molecules such as arjunolic acid, anthocyanins, apigenin, avicularin, berberine, baicalein, caffeic acid, gingerol, ginsenosides, calceolarioside, cannabidiol, carotenoids, chrysin, catechins, chrysoeriol, curcumin, eugenol, frederine, diosgenin, hesperidin, and kaempferol have all been reported to positively mitigate doxorubicin-mediated cardiotoxicity [88]. However, ours is the first to report the mitigating effect of plant extracts and indeed *Clerodendrum volubile* leaf and *Irvingia gabonensis* seed extracts against *TZM*-induced cardiotoxicity. Plant secondary metabolites especially polyphenols such as flavonoids, epicatechin, catechin, anthocyanidins, epigallocatechin gallate, carotenoids, terpenoids, sesquiterpenoids, and unsaturated fatty acids have been reported to protect against the deleterious effects of oxidative stress, reduce blood pressure, and improve endothelial dysfunction through several mechanisms [89, 90] which include activation of eNOS and reduced endothelial ET-1 secretion which are key in NO/cGMP pathway [91–95], as well as through activation of Akt/eNOS pathway [96]. Proanthocyanidins are also known to possess antithrombotic properties that are associated with endothelial protection and inhibition of inflammatory cells adhesion because it decreases P-selectin expression, thus, inhibiting leucocyte recruitment and thrombosis [96–98]. Proanthocyanidins are also known to have anti-inflammatory and antioxidant effects and improve circulating HDL-c levels without causing dyslipidemia, thus, exhibiting endothelium-protective, antiatherogenic, and cardioprotective activities [97, 99, 100]. Although coronary artery microthrombi formation was observed histopathologically in the rat hearts intoxicated with *TZM* but this was for profoundly improved with repeated oral *CVE* and *IGE* pretreatments with coronary artery revascularization observed in rat heart pretreated with *IGE*. *CVE* and *IGE* have reported to be abundantly rich in polyphenols and have been attributed to responsible for the high antioxidant activities of the plants [9, 16, 17, 25, 27]. Thus, the presence of polyphenols in high amounts in these extracts could be responsible for the observed cardioprotection offered against *TZM* cardiotoxicity. Similarly, oleanolic acid has been reported to be abundantly present in *CVE* and *IGE* and is known to decrease oxidative stress, apoptosis, and proteasomal activity following ischemia-reperfusion injury [101], antihyperlipidemic, and cardioprotective effects [23, 102]. Thus, the presence of this oil and other secondary metabolites could have also contributed to the cardioprotection offered by these extracts.

The clinical use of antioxidants in recent years has gained considerable interest. Epidemiological studies have suggested that diets (fruits and vegetables) that are richly high in antioxidant contents including vitamins A, C, and E and other phenolic contents might help decrease the risk of cardiovascular diseases (such as atherosclerosis, preeclampsia, or hypertension) and other chronic noncommunicable diseases such as diabetes mellitus, whose etiopathogenesis are thought to be mediated by oxidative stress [103]. Similarly, antioxidants have been documented to have useful clinical application in ameliorating drugs and xenobiotic toxicity. Drug, xenobiotic and environmental pollutant biotransformation results in the overproduction of free radicals in the body leading to lipid peroxidation, oxidative stress, and oxidative damage [104]. The ROS, thus, generated either directly or indirectly through the mediation of oxidative and inflammatory signals, disrupt the cellular equilibrium, and cause mitogenesis, mutagenesis, genotoxicity, and cytotoxicity and form the underlying pathophysiology for diseases such as diabetes, hypertension, atherosclerosis, cancer, Parkinsonism, and Alzheimer's disease [104]. However, studies have shown the benefit of antioxidants in protection against drug- and xenobiotic-induced toxicities. For example, the beneficial role of citrus fruit-derived flavonoid (diosmin) in ameliorating and preventing methotrexate-induced oxidative and inflammatory markers, suggesting the promising protective role of diosmin against methotrexate-induced toxicities in patients with cancer and autoimmune diseases have been reported [105]. Similarly, the protective effects of green tea (*Camellia sinensis*) on nicotine exposure-induced oxidative damage in mice leading to behavioral alterations including physical development, neuromotor maturation, and behavioral performance in newborn male and female mice have been demonstrated [106]. In another study, the cardioprotective role of the flavonoid and phenolic contents of *Murraya koenigii* (L.) Spreng. leaf extract against doxorubicin-induced cardiotoxicity in rat model was reported, indicating the protective potential of *Murraya koenigii* (L.) Spreng. leaf extract as an adjuvant therapy with doxorubicin [107]. Thus, in line with the above, the flavonoid and phenolic contents in *CVE* and *IGE* could be useful adjuvant therapy to ameliorate *TZM*-mediated cardiotoxicity.

The chemopreventive role of the standard antioxidant drug, vitamin C, in doxorubicin/trastuzumab-mediated cardiotoxicity which are primarily mediated via reactive oxidative stress, nitrosative stress, and inflammatory pathways is well documented (Fujita et al., 1982; Shimpo et al., 1991; Vincent et al., 2013; Akolkar et al., 2017; Singh et al., 2018; Carrasco et al., 2020) [108–113]. Vitamin C and its derivatives were reported to prevent myocardial lipoperoxidation and subsequent doxorubicin-mediated cardiomyopathy, thus, prolonged the life expectancy of experimental animals treated with doxorubicin [108, 109]. Vitamin C was also reported to mediate its cardioprotection via multimodal mechanisms which include reduced protein carbonyl formation, NOS activity, protein nitrosylation, iNOS expression, expression of apoptotic proteins (*Bax*, Bnip-3, *Bak*, and caspase-3), as well as decreased cardiac TNF-*α*, IL-1*β*, and IL-6 levels and increased Vitamin C transporter proteins (SVCT-2 and GLUT-4) [114]. Thus, the results of this study are in complete agreement with those of earlier studies where vitamin C pretreatments either prevented or ameliorated the deleterious effects of *TZM*-induced myocardial cellular oxidative damage.

Another notable finding of this study is the effect of *TZM* and the oral pretreatments with *CVE*, *IGE*, and Vit. C. *TZM*, unlike anthracycline cytotoxic agents, have been reported not to alter the lipid profile of cancer patients on it although preexisting diabetes mellitus, dyslipidemia, and obesity along with a number of cardiovascular risk factors and comorbidities are known to increase the propensity for cardiotoxicity in cancer patients on anthracycline/*TZM* therapy (Jawa et al., 2016; Kosalka et al., 2019; Abdel-Rasaq et al., 2019; Georgiadis et al., 2020) [115–118]. Going by the fact that repeated *TZM* injections did not significantly alter the complete lipids profile including the cardiovascular disease risk indices including AI and CRI of treated rats strongly indicated our result to be in tandem with earlier studies. AI is known to be a strong, reliable, and independent predictor of ischemic heart diseases including coronary artery disease and acute myocardial infarction (Cai et al., 2017; Kazemi et al., 2018; Gómez-Álvarez et al., 2020) [119–121]. AI is known to be a better predictor of coronary artery disease than traditional lipid parameters and other lipid ratios such as CRI and lipoprotein combined index (Cai et al., 2017) [119]. AI also reflects the lipid-driven inflammatory state in acute coronary syndrome (Zhan et al., 2016) [122]. The mere fact that *TZM* did not alter the value of this predictor is an indication that *TZM* does not mediate its cardiac dysfunction via the atherogenic mechanism. Similarly, this further strengthens the fact that *CVE* and *IGE* possess cardioprotective potentials.

## 5. Conclusion

Overall, results of our study for the first time showed that *CVE* and *IGE* effectively attenuated *TZM*-induced cardiotoxicity and their cardioprotective activities were mediated via antioxidant, free radical scavenging, antilipoperoxidation mechanisms although their antithrombotic mechanism remains plausible but more studies are required in this direction.

## Figures and Tables

**Table 1:** Group treatment of rats.

Groups	Treatments
Group I	10 ml/kg/day of sterile water *p.o.* for 7 days + 1 ml/kg/day of sterile water given *i.p.* for 7 days
Group II	400 mg/kg/day of *CVE* dissolved in 5% DMSO-sterile water *p.o.* for 7 days + 1 ml/kg/day of sterile water given *i.p.* for 7 days
Group III	400 mg/kg/day of *IGE* dissolved in 5% DMSO-sterile water *p.o.* for 7 days + 1 ml/kg/day of sterile water given *i.p.* for 7 days
Group IV	10 ml/kg/day of sterile water *p.o.* for 7 days + 2.25 mg/kg/day of *TZM* dissolved in sterile water given *i.p.* for 7 days
Group V	20 mg/kg/day of vitamin C dissolved in sterile water *p.o.* for 7 days + 2.25 mg/kg/day of *TZM* dissolved in sterile water given *i.p.* for 7 days
Group VI	400 mg/kg/day of *CVE* dissolved in 5% DMSO-sterile water *p.o.* for 7 days + 2.25 mg/kg/day of *TZM* dissolved in sterile water given *i.p.* for 7 days
Group VII	400 mg/kg/day of *IGE* dissolved in 5% DMSO-sterile water *p.o.* for 7 days + 2.25 mg/kg/day of *TZM* dissolved in sterile water given *i.p.* for 7 days

**Table 2:** Effect of repeated oral pretreatments with 400 mg/kg/day of *CVE* and *IGE* on the average body weights on days 1 and 7, percentage change in weight (% ∆wt.) and relative heart weight (RHW) of *TZM*-treated rats.

Group	Day 1 bwt. (g)	Day 7 bwt. (g)	% ∆wt.	RHW
I	175.8 ± 25.2	183.9 ± 20.5	05.1 ± 04.9	0.25 ± 0.01
II	178.2 ± 27.9	189.9 ± 34.4	06.2 ± 05.1	0.30 ± 0.02
III	183.4 ± 37.7	190.0 ± 39.9	03.5 ± 02.9	0.36 ± 0.04
IV	177.1 ± 20.4	188.5 ± 23.6	06.4 ± 02.6	0.37 ± 0.01
V	176.2 ± 20.5	185.0 ± 23.5	06.0 ± 05.4	0.38 ± 0.02
VI	171.5 ± 17.7	178.4 ± 17.2	04.2 ± 04.1	0.34 ± 0.02
VII	171.5 ± 21.4	180.7 ± 22.9	04.0 ± 04.3	0.40 ± 0.03

**Table 3:** Effect of 400 mg/kg/day of *CVE* and *IGE* on serum LDH and *cTn I* in *TZM*-intoxicated rats.

Treatment groups	LDH (U/L)	*cTn I* (ng/ml)
I	2826 ± 637.1	04.46 ± 01.04
II	3733 ± 365.0	05.05 ± 01.38
III	3634 ± 318.8	05.23 ± 01.26
IV	7200 ± 371.7^c+^	83.86 ± 13.04^c+^
V	2813 ± 344.4^c-^	11.06 ± 02.50^b-^
VI	3483 ± 310.9^c-^	06.35 ± 02.05^c-^
VII	3104 ± 405.0^c-^	04.45 ± 02.73^c-^

^c+^ represents a significant increase at *p* < 0.0001 when compared to Groups I-III values while ^b-^ and ^c-^ represent significant decreases at *p* < 0.001 and *p* < 0.0001, respectively, when compared to untreated positive (*TZM* only-treated only) control values, respectively.

**Table 4:** Effect of 400 mg/kg/day of *CVE* and *IGE* on serum lipid profile of *TZM*-treated rats.

Groups	Serum lipids				
	TG (mmol/l)	TC (mmol/l)	HDL-c (mmol/l)	LDL-c (mmol/l)	VLDC-c (mmol/l)
I	1.00 ± 0.11	1.37 ± 0.11	0.40 ± 0.03	0.51 ± 0.10	0.45 ± 0.05
II	0.79 ± 0.06	1.41 ± 0.13	0.41 ± 0.04	0.64 ± 0.08	0.36 ± 0.04
III	0.79 ± 0.09	1.47 ± 0.12	0.44 ± 0.04	0.67 ± 0.09	0.36 ± 0.04
IV	0.96 ± 0.05	1.53 ± 0.09	0.44 ± 0.02	0.66 ± 0.09	0.43 ± 0.02
V	0.94 ± 0.10	1.51 ± 0.10	0.44 ± 0.02	0.64 ± 0.07	0.43 ± 0.04
VI	0.86 ± 0.09	1.40 ± 0.13	0.40 ± 0.03	0.62 ± 0.07	0.39 ± 0.04
VII	0.80 ± 0.06	1.45 ± 0.08	0.42 ± 0.03	0.67 ± 0.04	0.36 ± 0.03

**Table 5:** Effect of 400 mg/kg/day of *CVE* and *IGE* on atherogenic index (AI) and coronary artery disease index (CRI) in *TZM*-intoxicated rats.

Treatment groups	AI	CRI
I	01.19 ± 0.17	03.39 ± 0.08
II	01.53 ± 0.13	03.45 ± 0.11
III	01.34 ± 0.22	03.39 ± 0.04
IV	01.65 ± 0.16	03.52 ± 0.16
V	01.18 ± 0.06	03.42 ± 0.10
VI	01.42 ± 0.10	03.50 ± 0.09
VII	01.59 ± 0.09	03.45 ± 0.07

**Table 6:** Antioxidant activities of 400 mg/kg/day of *CVE* and *IGE* in *TZM*-intoxicated rat cardiac tissue.

Groups	Antioxidant parameters					
	GSH	GST	GPx	SOD	CAT	MDA
I	26.8 ± 3.0	31.7 ± 1.1	28.5 ± 2.8	08.4 ± 0.6	44.5 ± 1.2	0.4 ± 0.1
II	35.0 ± 3.6	29.3 ± 0.9	32.5 ± 3.3	07.7 ± 0.5	42.0 ± 6.8	0.5 ± 0.3
III	33.5 ± 4.5	22.9 ± 1.7	24.8 ± 1.8	06.2 ± 0.9	33.4 ± 7.2	0.5 ± 0.1
IV	16.7 ± 2.1^c-^	19.8 ± 2.2^c-^	46.9 ± 2.0^f+^	03.6 ± 0.2^c-^	17.7 ± 2.4^c-^	0.8 ± 0.1^f+^
V	29.5 ± 3.3^b+^	24.7 ± 0.6^b+^	19.9 ± 1.1^f-^	06.5 ± 0.7^c+^	26.0 ± 2.6^b+^	0.5 ± 0.1^f-^
VI	28.3 ± 1.6^b+^	25.0 ± 0.5^b+^	19.6 ± 1.8^f-^	08.1 ± 0.6^c+^	26.9 ± 1.2^b+^	0.4 ± 0.1^f-^
VII	34.8 ± 2.7^c+^	26.4 ± 0.5^c+^	16.7 ± 2.1^f-^	07.6 ± 0.7^c+^	30.2 ± 2.6^c+^	0.5 ± 0.1^f-^

^c-^ represents a significant decrease at *p* < 0.0001 when compared to Groups I-III (controls) values while ^f+^ represents a significant increases at *p* < 0.0001 when compared to Groups I-III values; ^b+^ and ^c+^ represent significant increases at *p* < 0.05 and *p* < 0.0001, respectively, when compared to Groups IV values while ^f-^ represents a significant decrease at *p* < 0.0001 when compared to untreated positive control (*TZM* treated only, Group IV).

## Data Availability

Answer: Yes. Comment.

## Abbreviations

Akt/eNOS: Akt-dependent phosphorylation of endothelial nitric oxide synthase
AI: Atherogenic index
AST: Aspartate transaminase
*Bak*: B-cell associated k protein
*Bax*: B-cell associated x protein
Bnip3: Bcl-2 adenovirus E1B 19 kDa-interacting protein 3
CAT: Catalase
CRI: Coronary artery index
*cTn I*: Cardiac troponin I
*CVE*: *Clerodendrum volubile* ethanol leaf extract
DMSO: Dimethyl sulfoxide
DPPH: 1,1-diphenyl-2-picrylhydrazyl
DTNB: 5,5-dithiobisnitro benzoic acid
eNOS: Endothelial nitric oxide synthase
ET-1: Endothelin-1
GLUT-4: Glucose transporter protein-4
GPx: Glutathione peroxidase
GSH: Reduced glutathione
GST: Glutathione S-transferase
HCl: Hydrochloric acid
HDL-c: High-density lipoprotein cholesterol
*IGE*: *Irvingia gabonensis* ethanol seed extract
IL-1*β*: Interleukin-1 beta
IL-6: Interleukin-6
iNOS: Induced nitric oxide synthase
*i.p.*: Intraperitoneal
KCl: Potassium chloride
LDH: Lactate dehydrogenase
LDL-c: Low-density lipoprotein cholesterol
MDA: Malondialdehyde
*mPTP*: Mitochondrial permeability transition pore
NO/cGMP: Nitric oxide-cyclic guanosine monophosphate
NOS: Nitric oxide synthase
*p.o.*: *Per os*
% ∆wt.: Percentage change in weight
RHW: Relative heart weight
ROS: Reactive oxygen species
S.D.: Standard deviation of the mean
S.E.M.: Standard error of the mean
SOD: Superoxidase dismutase
SVCT-2: Sodium-dependent vitamin C cotransporter isoform 2
TBA: Thiobarbituric acid
TC: Total cholesterol
TCA: Tricarboxylic acid
TG: Triglyceride
TNF-*α*: Tumor necrosis factor-alpha
*TZM*: Trastuzumab (r-DNA origin)
UNILORIN: University of Ilorin
UV: Ultraviolet
Vit. C: Vitamin C
VLDL-c: Very low-density lipoprotein cholesterol.
